# Modulation of CXCR4, CXCL12, and Tumor Cell Invasion Potential In Vitro by Phytochemicals

**DOI:** 10.1155/2009/491985

**Published:** 2009-03-24

**Authors:** Erin L. Hsu, Natalie Chen, Aya Westbrook, Feng Wang, Ruixue Zhang, Robert T. Taylor, Oliver Hankinson

**Affiliations:** ^1^Molecular Toxicology Interdepartmental Doctoral Program, University of California Los Angeles, Los Angeles, CA 90095, USA; ^2^Department of Pathology and Laboratory Medicine, University of California Los Angeles, Los Angeles, CA 90095, USA; ^3^Feinberg School of Medicine, Northwestern University, 303 E. Chicago Avenue Ward 10-258, Chicago, IL 60611, USA; ^4^Jonsson Comprehensive Cancer Center, University of California Los Angeles, Los Angeles, CA 90095, USA; ^5^Department of Hepatic Biology, CellzDirect Invitrogen Corporation, 1624 Headway Circle, Austin, TX 78754, USA

## Abstract

CXCR4 is a chemokine receptor frequently overexpressed on primary tumor cells. Organs to which these cancers metastasize secrete CXCL12, the unique ligand for CXCR4, which stimulates invasion and metastasis to these sites. Similar to our previous work with the chemoprotective phytochemical, 3,3′-diindolylmethane (DIM), we show here that genistein also downregulates CXCR4 and CXCL12 and subsequently lowers the migratory and invasive potentials of breast and ovarian cancer cells. Moreover, genistein and DIM elicit a significantly greater cumulative effect in lowering CXCR4 and CXCL12 levels than either compound alone. Our data suggest a novel mechanism for the protective effects of phytochemicals against cancer progression and indicate that in combination, these compounds may prove even more efficacious.

## 1. Introduction

Phytochemicals have long been implicated in a protective effect for cancer. Soybean- and cruciferous vegetable-enriched diets seem to be particularly protective, but the mechanisms for this anticarcinogenicity are not fully understood. Genistein, a dietary phytoestrogen belonging to the isoflavone class of flavonoids, is thought to have anticarcinogenic activities, particularly for breast and prostate cancer [1, 2]. Dietary soy has been shown in mice to inhibit prostate tumor growth through inhibition of cell proliferation, increased apoptosis, and reduced microvessel density [3]. Epidemiology studies of Asian women indicate that consumption of a traditional diet high in soy confers significant protection against breast cancer [4].

3,3′-diindolylmethane (DIM), a breakdown product of glucobrassicins, which are found in cruciferous vegetables, has been shown in vivo to have protective effects for breast cancer [5]. We have shown previously that DIM lowers the levels of CXCR4 and CXCL12, a chemokine receptor and its unique ligand required for the metastasis of breast cancer [6, 7]. In addition to mediating the directional homing of primary breast cancer cells to secondary organ sites, CXCR4 and CXCL12 are important in other aspects of cancer progression, such as adhesion, proliferation, and angiogenesis. These effects are not limited to breast cancer, as the interaction between CXCR4 and CXCL12 is implicated in the progression of many different types of cancer.

The biological effects of genistein are extensive and include antioxidant activity, weak estrogenic/antiestrogenic activity, upregulation of apoptosis, inhibition of angiogenesis, inhibition of DNA topoisomerase II, and inhibition of protein tyrosine kinases (PTKs). Genistein has been shown to regulate specific sex steroid receptors, inhibit NFkB, downregulate TGF-*β*, and inhibit EFG-stimulated growth. Furthermore, genistein inhibits 2,3,7,8-tetrachlorodibenzo-*p*-dioxin (dioxin)-induced CYP1A1 activity, and isoflavones can prevent the CYP1A1-mediated binding of benzo[a]pyrene (B[a]P) metabolites to DNA [8].

Our previous observations that DIM downregulates CXCR4 and CXCL12 in breast and ovarian cancer cells represent a novel mechanism for the chemoprotective effects of this phytochemical. Here, we demonstrate that these effects are not unique to DIM, but can also be seen with genistein. Interestingly, we see that the combined effect of DIM and genistein elicits a greater downregulation of CXCR4 and CXCL12 than either compound alone, indicating that the phytochemicals used in combination may be even more potent in their chemoprotective properties. We also demonstrate that like DIM, genistein specifically inhibits chemotaxis and chemoinvasion of breast and ovarian cancer cells toward CXCL12 in vitro.

## 2. Materials and Methods

### 2.1. Chemical Reagents and Cell Culture

Genistein was purchased from Sigma (St. Louis, Mo, USA), DIM was purchased from LKT Laboratories (St. Paul, Minn, USA), and the remaining phytochemicals were a kind gift from Dr. David Heber (University of California, Los Angeles, Calif, USA). BG-1 cells were generously provided by Dr. Kenneth Korach (National Institute of Environmental Health Sciences, NC, USA). MCF-7 and MDA-MB-231 cells were purchased from the American Type Culture Collection (ATCC, Manassas, Va, USA). MCF-7 and BG-1 cells were maintained in Minimal Essential Medium. MDA-MB-231 cells were maintained in Dulbecco's Modified Eagle's Medium containing 4 mM L-glutamine. All media were purchased from Invitrogen (Carlsbad, Calif, USA) and supplemented with 10% fetal bovine serum (FBS; Omega, Tarzana, Calif, USA), 100 U/mL penicillin/100 *μ*g/mL streptomycin solution (Gemini Bio-Products, West Sacramento, Calif, USA), and 0.25 U/mL Amphotericin B (Omega). Cells were maintained at 37°C under 5% CO_2_. For all experiments, chemicals were dissolved in DMSO and administered to cells with a final concentration of DMSO at 0.1% in the medium.

### 2.2. RNA Extraction, cDNA Synthesis, and Real-Time PCR

RNA isolation, cDNA synthesis, and taqman multiplex real-time PCR were performed as previously described [6]. CXCR4 and CXCL12 cDNAs were amplified using *Assays on Demand* (Affinity Bioreagents, Golden, Colo, USA; product numbers Hs00607978_s1 and Hs00171022_m1, resp.; sequences proprietary). Quantities were normalized to those for the 36B4 ribosomal housekeeping gene. The forward and reverse primers used for 36B4 quantification were 5′-CCACGGTGCTGAACATGCT-3′ and 5′-TCGAACACCTGCTGGATGAC-3′, respectively. The 36B4 probe sequence was 5′-Texas Red-ACCATCTCCCCCTTCTCCTTTGGGCT-Iowa Black-3′. All primers and probes were purchased from Integrated DNA Technologies (Coralville, Iowa, USA). Real-time PCR was carried out using the ICycler IQ (BioRad, Hercules, Calif, USA) or 7500 Fast (Applied Biosystems, Foster City, Calif, USA) under standard protocols. Data were analyzed using the ICycler or ABI software and Microsoft Excel, and significance was evaluated using Student's *t*-test.

### 2.3. Flow Cytometry

For surface staining of CXCR4 and intracellular staining of CXCL12, cells were grown to 70% confluence and treated with genistein for 24 or 48 hours. In the case of CXCL12 quantification, the cells were also cotreated with 1 *μ*g/mL Brefeldin A (GlogiPlug; BD PharMingen, Franklin Lakes, NJ, USA) for the final six hours of incubation to inhibit protein secretion. Cells were harvested and stained as previously described [6, 9] with either primary CXCL12 antibody (R&D Cat. no. MAB350; Minneapolis, Minn, USA), primary CXCR4 antibody (Affinity Bioreagents Cat. no. OPA1-01101; Ill, USA) or the appropriate primary IgG isotype control antibody, followed by staining with either goat antimouse or antirabbit IgG-FITC secondary antibodies (BD-PharMingen and Caltag, Carlsbad, Calif, USA, resp.). Fluorescence was quantified using a FACScan analytic flow cytometer (Becton Dickinson, UCLA Flow Cytometry Core Facility). Data were analyzed using FCS Express3 Lite Software (DeNovo, Inc., Thornhill, ON, Canada).

### 2.4. Chemotaxis and Chemoinvasion Assays

Cells were pretreated with genistein, DIM, or the combination, followed by chemotaxis and invasion assays, performed as we have described previously [6]. After migration/invasion, MTS assays (Promega, Madison, Wis) were performed in each individual transwell to control for small variations in cell number. After detection of the formazan product at 490 nm, cells on the inserts were washed with PBS and those on the upper layer were gently removed with a prewet Q-tip. Cells on the lower layer were fixed in 100% methanol and stained with crystal violet. Membranes were manually excised from the inserts, mounted on microscope slides, and divided into 8 equal sections. Cells in one random viewing field from each section were counted at 40x magnification, and the average was calculated. Average counts from each insert were normalized to the equivalent MTS values. Data are expressed as chemotaxis or chemoinvasion indices, which were defined as the normalized number of cells in the experimental group relative to the control group. Statistical analyses were performed using a two-tailed Student's *t*-test.

## 3. Results

### 3.1. Genistein Downregulates CXCR4 and CXCL12 in Breast and Ovarian Cancer Cells

**Figure 1:**
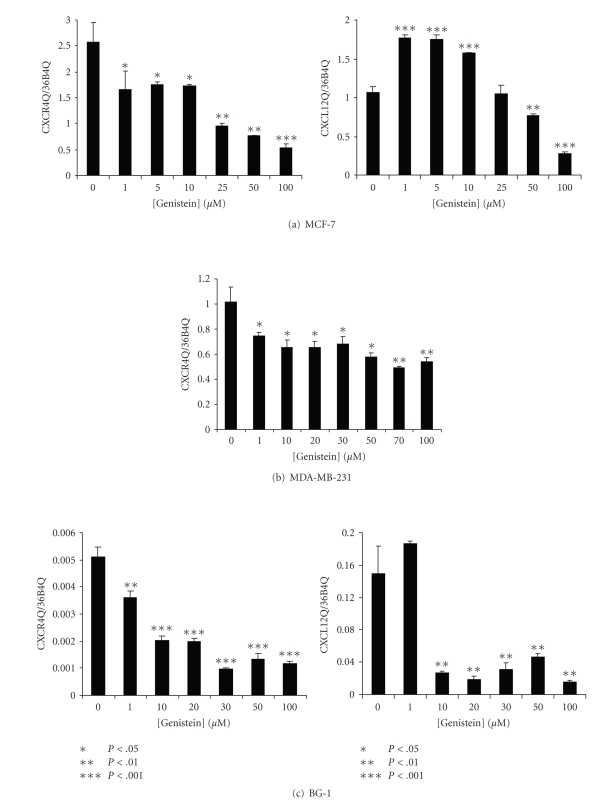
*Dose-response curves for CXCR4 and CXCL12 mRNAs after treatment of breast and ovarian cancer cells with 1–10 *μ*M genistein.* (a) CXCR4 and CXCL12 mRNA quantification after treatment of MCF-7 cells with increasing concentrations of genistein for 24 hours. (b) CXCR4 quantification after treatment of MDA-MB-231 cells with genistein for 24 hours. MDA-MB-231 cells do not express CXCL12 as determined by real-time PCR. (c) CXCR4 and CXCL12 quantification after treatment of BG-1 cells with increasing doses of genistein for 24 hours. All data were normalized to corresponding mRNA quantities for the 36B4 housekeeping gene. *P < .05, **P < .01, ***P < .001; relative to 0 *μ*M genistein control.

MCF-7 and MDA-MB-231 breast cancer cells and BG-1 ovarian cancer cells were treated with concentrations of genistein ranging from 1–100 *μ*M (at a constant final DMSO concentration) for 24 hours, after which time CXCR4 and CXCL12 mRNAs were quantified by real-time PCR. CXCR4 mRNA levels were significantly decreased at 1 *μ*M genistein in all three cell lines (Figures 1(a)–1(c)). Maximal downregulation was seen at 100 *μ*M, 50 *μ*M, and 30 *μ*M genistein in each cell line, respectively. Significant downregulation of CXCL12 mRNA occurred at a concentration of 50 *μ*M genistein in MCF-7 cells and 10 *μ*M genistein in BG-1 cells (Figures 1(a)–1(c)). Interestingly, low doses of genistein (1–10 *μ*M) significantly *increased* CXCL12 mRNA levels in MCF-7 cells, but had the opposite effect in BG-1 cells at these doses. MDA-MB-231 cells do not express detectable levels of CXCL12.

**Figure 2:**
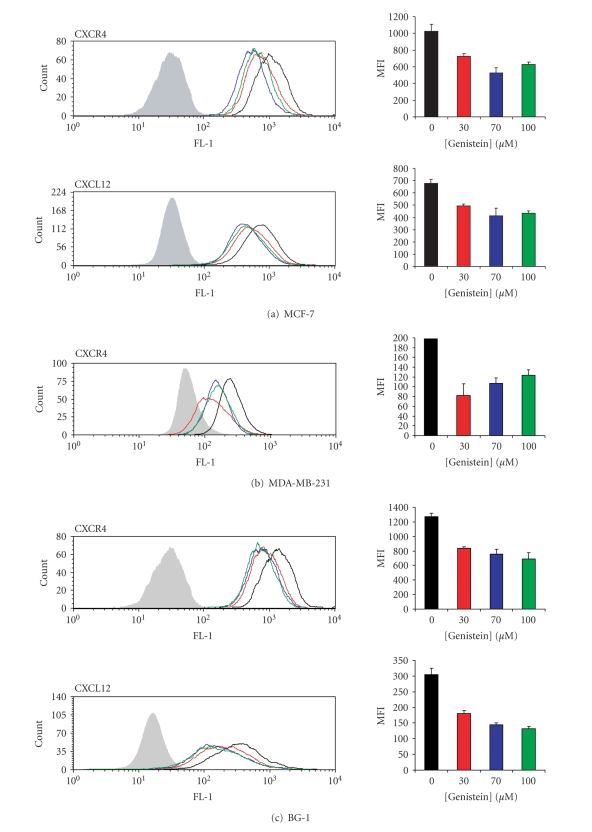
* Genistein downregulates CXCR4 surface expression and intracellular CXCL12 expression.* Downregulation of CXCR4 protein expression in MCF-7, MDA-MB-231, and BG-1 cells at concentrations of genistein ranging from 0–100 *μ*M for a 24-hour period (a)–(c). Downregulation of intracellular CXCL12 protein expression in (a) MCF-7 and (c) BG-1 cells at 30–100 *μ*M genistein. Fluorescence distribution plots depict one representative experiment. Isotype controls are represented by filled histograms. Mean fluorescence intensities (MFI) and their corresponding standard deviations are derived from three independent experiments.

Flow cytometric analysis was used to quantify surface CXCR4 and intracellular CXCL12 expression. Cells were treated with 30, 70, or 100 *μ*M genistein for 24 hours, harvested, stained, and analyzed for surface expression levels of CXCR4. Relative to DMSO-treated controls, CXCR4 was found to be downregulated by genistein in MCF-7, MDA-MB-231, and BG-1 cells at all three doses (Figures 2(a)–2(c)). Analysis of CXCL12 intracellular expression showed that relative to DMSO-treated control cells, CXCL12 levels were reduced by all three doses of genistein in both MCF-7 and BG-1 cells (Figures 2(a), 2(c)).

### 3.2. Downregulation of CXCR4 and CXCL12 Is Greater after Treatment with Both Genistein and DIM than with Either Compound Alone

**Figure 3:**
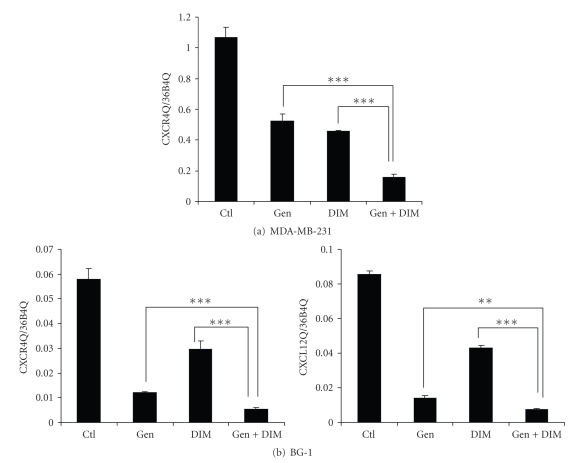
*
In combination, genistein and DIM are more efficacious in the downregulation of CXCR4 and CXCL12 mRNAs than either compound alone.* The effect of downregulation of CXCR4 in (a) MDA-MB-231 and (b) BG-1 cells by 20 *μ*M DIM and 100 *μ*M genistein in combination is greater than their individual effects. The same is seen in (b) BG-1 cells after CXCL12 quantification. Ctl = DMSO alone; **P < .01, ***P < .001.

We previously found that DIM downregulates CXCR4 and CXCL12 mRNAs and proteins in invasive breast and ovarian cancer cells [6]. We therefore tested whether the downregulation elicited by cotreatment with both DIM and genistein was additive or synergistic in these cell lines. Using real-time PCR, we quantified gene expression after treatment of MDA-MB-231 (CXCR4 only) or BG-1 (both CXCR4 and CXCL12) with 20 *μ*M DIM, 100 *μ*M genistein, or the combination. We found that in both cell lines, the degree of downregulation of CXCR4 was greater after treatment with both phytochemicals in combination (Figures 3(a)-3(b)). In MDA-MB-231 cells, DIM reduced levels of CXCR4 by 57%, genistein by 51%, and the combination by 85% (Figure 3(a)). In BG-1 cells, DIM downregulated CXCR4 by 49%, genistein by 79%, and DIM + genistein by 90%. Similarly, DIM lowered the levels of CXCL12 by 50%, genistein lowered levels by 84%, and genistein by 91% (Figure 3(b)). These effects cannot be attributed to cytotoxicity, since neither DIM, genistein, nor the combination was cytotoxic at these doses for the 24 hour time period (data not shown).

### 3.3. Genistein Specifically Inhibits Chemotaxis and Chemoinvasion of Breast and Ovarian Cancer Cells toward CXCL12

**Figure 4:**
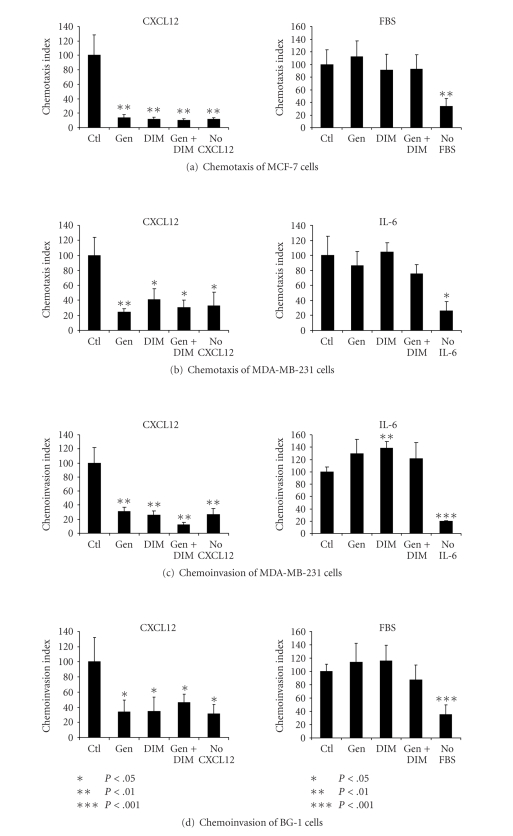
*DIM specifically inhibits chemotaxis and chemoinvasion of breast and ovarian cancer cells toward CXCL12.* (a) Quantification of chemotaxis of MCF-7 cells toward CXCL12 or FBS after treatment with DMSO alone (Ctl), 20 *μ*M DIM, 70 *μ*M genistein, or the combination of DIM + genistein for 24 hours. (b)-(c) Chemotaxis and chemoinvasion of MDA-MB-231 cells toward CXCL12 or IL-6 were quantified after pretreatment with DIM and/or genistein as in (a). (d) Quantification of chemoinvasion toward CXCL12 or FBS after treatment of BG-1 cells with DIM and/or genistein. *P < .05, **P < .01, ***P < .001; compared with control + chemoattractant.

Since CXCR4 and CXCL12 mediate directional migration, and since we saw previously that DIM inhibited chemotaxis and chemoinvasion of these cells, we performed assays to determine whether genistein could inhibit migration through fibronectin and/or invasion through matrigel. We found that pretreatment with genistein inhibited the directional migration of MCF-7 cells, with a resulting migration rate similar to the background rate (DMSO-treated cells exposed to no CXCL12 gradient, Figure 4(a)). However, we did not see a further reduction in migration after cotreatment with genistein and DIM, presumably because both chemicals used independently at these doses fully reduced migration to background levels. This result indicates that the degree to which genistein and DIM downregulate surface CXCR4 levels at these doses may be sufficient to significantly impact the homing of breast cancer cells to areas of high CXCL12 expression. As we have seen previously with DIM, the inhibition of chemotaxis by genistein specifically affects migration toward CXCL12, since an inhibition of MCF-7 cell chemotaxis toward FBS was not observed.

MCF-7 cells do not invade through matrigel (a synthetic extracellular matrix), and we were therefore unable to evaluate invasive potential in these cells. However, MDA-MB-231 breast cancer cells are invasive and we were able to quantify both chemotactic and chemoinvasive potential in this cell line. We found that genistein, DIM, and the combination significantly inhibited chemotaxis and chemoinvasion of these cells toward CXCL12, but not toward IL-6, a known in vitro chemoattractant for MDA-MB-231 cells (Figures 4(b)-4(c)). This result indicates that the inhibitory effects of DIM and genistein are specific for CXCL12-induced chemoattraction/chemoinvasion and is not merely a general effect on migration or invasion. A similar result was seen with BG-1 ovarian cancer cells, which are also invasive in vitro (Figure 4(d)). These cells do not migrate through matrigel toward IL-6 (data not shown), but did exhibit moderate chemoinvasion toward FBS.

## 4. Discussion

The antiestrogenic activity of genistein may mediate, in part, the protective effects of soy for breast and other cancers [10]. Although other nonestrogenic mechanisms of action such as PTK and topoisomerase II inhibition likely play a role in these protective effects, we describe here an additional novel mechanistic pathway in which genistein and possibly other phytochemicals may be protective. We have found that genistein downregulates CXCR4 in the ER-positive (ER+) breast cancer cell line, MCF-7, the ER-negative (ER−) breast cancer cell line, MDA-MB-231, and the ER+ ovarian cancer cell line, BG-1. Furthermore, CXCL12, the unique ligand for CXCR4, is downregulated by genistein in both MCF-7 and BG-1 cells. We show that this downregulation results in a subsequent inhibition of migration and invasion of these cells toward CXCL12 in vitro.

We found previously that DIM downregulates CXCR4 and CXCL12 in MCF-7, MDA-MB-231, and BG-1 cells [6]. We show here that when used in combination, the effects of DIM and genistein on CXCR4 and CXCL12 mRNA levels are greater than with either compound alone, suggesting that phytochemicals used in combination may increase the efficacy of their protective effects. At 20 *μ*M DIM and 70 *μ*M genistein, we did not find the combination to further inhibit chemotaxis or chemoinvasion of breast or ovarian cancer cells since these concentrations of the phytochemicals fully inhibit these processes. However, at the slightly lower doses of 10 *μ*M DIM and 50 *μ*M genistein, the combination does appear to be more effective at inhibiting chemotaxis of MDA-MB-231 cells. At these latter doses, the individual phytochemicals did not fully reduce chemotaxis of these cells to background levels. These results therefore suggest that lower doses of the phytochemicals used in combination may be equally or more effective in chemoprotection than a higher dose of a single phytochemical.

The mechanisms of action of DIM and genistein in the downregulation of CXCR4 and CXCL12 remain to be determined. DIM and genistein both act as weak agonists/antagonists of the estrogen receptor, and it is therefore possible that the compounds can act through the same mechanism. Importantly however, we see downregulation of CXCR4 and a subsequent inhibition of chemotaxis and chemoinvasion in MDA-MB-231 cells, which do not express the estrogen receptor. On the other hand, DIM is a ligand for the AHR, whereas genistein has not been shown to bind the AHR in the cell lines we used. Genistein is a specific PTK inhibitor as well as an inhibitor of topoisomerase II, and may modulate TGF-*β* signal transduction [11, 12]. Interestingly, both genistein and DIM are known to inhibit NFkB in breast, prostate, and pancreatic cancer cells [13–15]. Since CXCR4 is regulated at the transcriptional level by NFkB, it is plausible that the mechanism of CXCR4 and/or CXCL12 downregulation by DIM and/or genistein could be through NFkB inactivation or downregulation [16, 17].

Condoning the general use of genistein as a supplement is at this point controversial. Soy products are extensively consumed in Asian populations without apparent adverse effects, but experimental data have led to concerns about the safety of genistein and other constituents of soy. Although it was well tolerated, high doses of genistein in chronic studies caused an increase in the weights of the kidney, spleen, adrenal, and testes in male rats and an increase in liver, kidney, spleen, ovary, and uterus weights in female rats [18]. In the same study, histological changes were seen in the reproductive organs of both male and female rats. These findings were attributed to the estrogenic properties of genistein. Genistein can behave as both an estrogen and an antiestrogen, and the net estrogenic effect of the chemical has proven difficult to quantify [11]. A chronic exposure study very recently carried out by the National Toxicology Program showed a significant increase in the incidence of mammary gland adenoma and adenocarcinoma (combined) [19]. Importantly, the time of administration appears to significantly impact whether genistein elicits a protective, adverse, or no effect [20]. For example, dimethylbenzanthracene (DMBA)-induced mammary cancer was reduced after prepubertal and combined prepubertal and adult administration of genistein, but not after prenatal-only or adult-only treatments [2].

Dose also plays a critical role in the biological effects of genistein; although doses greater than 10 *μ*M over an extended period of time inhibit the growth of both ER+ and ER− breast cancer cells, low doses of genistein (<1 *μ*M) appear to *stimulate* the growth of ER+ breast cancer cells [21, 22]. Interestingly, CXCL12 has been shown to mediate the proliferative effects of estradiol in breast cancer cells [23]. We noted a significant increase in CXCL12 mRNA after treatment with low doses (1–10 *μ*M) of genistein in MCF-7 cells, although this effect was not seen in MDA-MB-231 or BG-1 cells. An upregulation of CXCL12 by low doses of genistein remains to be confirmed in tissues that serve as common sites of metastasis such as the lung and bone. However, in light of what is known about the toxic and potentially carcinogenic effects of genistein, this observation underscores the importance of thorough safety analyses prior to condoning the use of phytochemicals as dietary supplements. In particular, it highlights the importance of dose, especially at the tissue level, when assessing the impact of genistein on the development and progression of breast and other cancers.

The doses of genistein and DIM described here are likely achievable in humans upon supplementation, especially at the tissue level. Total genistein plasma concentrations of up to 20 *μ*M were obtained after feeding human volunteers a genistein supplement [24–26]. Furthermore, genistein was found to accumulate in certain organs to considerably higher concentrations [27], and this is likely to be the case for fatty tissues, such as the breast, in particular. When mice were administered a single oral dose of DIM, a serum concentration of approximately 5 *μ*M was achieved. Much higher concentrations accumulated in certain organs [28]. Thus, the concentrations of genistein and DIM that we have used in our studies are likely to reflect attainable doses in the human.

Since CXCR4 and CXCL12 are implicated in the progression of many different cancers, it is of interest to determine whether DIM and/or genistein downregulate these proteins in cancer cell lines of other origins. It will also be important to investigate whether CXCL12 is downregulated by phytochemicals in tissues that serve as preferred sites of metastasis for these cancers, such as the lung and bone. Furthermore, since many other phytochemicals have been implicated in cancer protection, it will be prudent to determine whether phytochemicals other than DIM and genistein exert similar effects on CXCR4 and CXCL12 levels. Finally, phytochemicals in combination should be tested in vivo to determine whether a potentiated effect can be achieved.

## 5. Conclusion

DIM has been suggested as a potential chemotherapeutic for ER+ breast cancers. Surprisingly however, we have found that DIM and genistein downregulate CXCR4 and CXCL12 in both ER+ and ER− cell lines, indicating that these phytochemicals may be effective in the treatment of both early- and late-stage breast cancers. Effective therapies for advanced disease are lacking, and the potential use of compounds as innocuous as phytochemicals for treatment of either early- or late-stage cancers is an attractive alternative. Furthermore, the increased response of CXCR4 and CXCL12 downregulation by DIM and genistein in combination may prove useful in eliciting a potentiated effect in vivo and perhaps allow for optimization of the associated biological responses.
